# Echoes of Inequity: A Critical Examination of the Past, Present, and Future of Cardiac Health Equity

**DOI:** 10.1007/s11886-025-02275-y

**Published:** 2025-09-19

**Authors:** Toluwalase Awoyemi, Cedrick Mutebi, Quentin R. Youmans, Ike S. Okwuosa, Clyde W. Yancy, Kamari Ositelu

**Affiliations:** 1 Feinberg School of Medicine, Northwestern University, Chicago, IL USA; 2 Division of Cardiology, Department of Medicine, Northwestern University Feinberg School of Medicine, 676 North Saint Claire Street, Arkes 600, Chicago, IL 60611 USA

**Keywords:** Health equity, Social determinants of health, Social epidemiology, Heart failure, Coronary artery disease, Implementation science

## Abstract

**Purpose of review:**

This review explores the historical, structural, and biological foundations of cardiovascular (CV) health inequities in the U.S. It examines how disparities by ancestry, sex, geography, income, immigration status, and race have emerged, persisted, and, in some cases, worsened while evaluating strategies for advancing equity.

**Recent findings:**

Despite progress in prevention and treatment, key disparities remain entrenched. Structural inequities, socioeconomic exclusion, and underrepresentation in research continue to shape outcomes. Social adversity is increasingly understood to exert biological effects through mechanisms such as chronic stress, cardio-kidney-metabolic dysfunction, and epigenetic aging. Novel tools, including place-based deprivation indices, precision risk prediction models, and community-driven interventions offer actionable pathways forward but remain underutilized or unevenly implemented.

**Summary:**

Cardiac health equity requires more than clinical innovation; it demands structural reform, inclusive science, and equity-centered implementation. Future solutions must embed social context into care, research, and policy to drive durable, population-level impact.

**Supplementary Information:**

The online version contains supplementary material available at 10.1007/s11886-025-02275-y.

## Introduction: What is Health Equity?

Cardiovascular disease (CVD) remains the leading cause of death in the United States (U.S.) and across the globe, yet the burden is not equally shared. Efforts to advance health equity in the U. S. gained national attention with the 1985 Heckler Report, the first government-issued document to comprehensively examine racial and ethnic disparities [1]. The report identified six leading causes of death that accounted for over 80% of excess mortality among minority populations, with CVD ranking as the second most significant contributor. At the time, it attributed approximately 60,000 excess deaths annually to systemic, avoidable, and unjust social conditions, setting the original bar for health inequities [1]. This landmark report shifted the lens from health outcomes attributed to solely individual behaviors to a broader set of social and structural determinants.

Health inequities are especially pronounced across the continuum of cardiovascular health, from prevention and diagnosis to treatment and outcomes. Social and structural barriers significantly impact how certain groups receive cardiovascular care. Health equity provides a conceptual framework for understanding and addressing these disparities. The Robert Wood Johnson Foundation defines health equity as “a fair and just opportunity to be as healthy as possible” [2]*.* Health equity aims to systematically identify these health inequities, implement policy and practice modifications to mitigate them, evaluate the efficacy of interventions, and continually refine approaches based on evidence. The aim of this critical review is to analyze how the conceptualization of health equity has evolved respective to cardiovascular health disparities and the future directions needed to foster cardiovascular health equity.

## Disparities That Have Worsened

Disparities related to socioeconomic status and race have widened in recent decades. One of the most concerning trends is the widening income-based gap in predicted CVD risk. From 1988–1994 to 2015–2018, the relative difference in 10-year predicted CVD risk between individuals in the lowest income group (PIR < 1) and those in the highest income group (PIR 5) increased markedly, from 6 to 70% [3]. Notably, while higher-income groups saw significant reductions in CVD risk, the lowest-income group experienced no meaningful change, thereby exacerbating relative disparities.

This pattern is echoed in broader analyses from 1988 to 2018, which showed that cardiovascular health gains were unequally distributed. Individuals in the lowest income group experienced little to no improvement, whereas those in higher income groups saw substantial benefits [4]. Regarding race, while the use of effective therapies in HF has improved survival overall, mortality disparities among African American patients have worsened, reflecting unequal access to and/or benefit from contemporary treatment options [5].

## Disparities That Have Persisted

Several cardiovascular disparities have remained relatively unchanged, indicating the presence of entrenched structural barriers. Despite improvements in the national achievement index, individuals in the lowest income strata continue to bear a disproportionate burden of adverse outcomes [3]. Likewise, although HF outcomes have improved overall, disparities in mortality persist by age, race, and geography, particularly in nonmetropolitan areas [6].

Racial and ethnic disparities in Atherosclerotic Cardiovascular Disease (ASCVD) treatment uptake also remain. While statin use has increased across all groups, gaps in the adoption of other evidence-based therapies limit the full benefits of advancements in care [7]. Additionally, although the Black–White cardiovascular mortality gap has narrowed since 1999, Black individuals, especially younger adults, continue to face higher mortality rates. These disparities are most pronounced in regions with high levels of racial segregation, emphasizing the ongoing impact of structural racism on health outcomes [6].

## Disparities That Have Improved

Despite persistent inequities, cardiovascular health has shown overall improvement. Between 1988–1994 and 2015–2018, the mean 10-year predicted CVD risk in the general population declined from 7.8% to 6.4%, reflecting progress in prevention, early detection, and treatment [3]. In heart failure (HF) specifically, age-adjusted mortality rates among women fell substantially from 1999 to 2020 [8].

Among older adults hospitalized for HF between 2004 and 2018, inpatient mortality and healthcare costs decreased despite rising comorbidity burdens [6]. Overall, cardiovascular mortality rates declined from 1999 to 2019 for both Black and White adults, with the absolute mortality gap between the two groups narrowing over time [6]. Additionally, lipid management has advanced: between 1999 and 2020, both total cholesterol control and statin use has improved across racial and ethnic groups lowering atherosclerotic cardiovascular disease (ASCVD) [7].

## Health Inequity as a Function of Rurality

Adults residing in U.S. census-defined rural counties make up 20% of the population and have been shown to have higher age-standardized rates of cardiovascular disease (CVD) risk factors: hypertension (37.1% vs. 30.9%), hyperlipidemia (29.3% vs. 26.7%), obesity (41.1% vs. 30.0%), and diabetes (11.2% vs. 9.8%) when compared to their urban counterparts [9]. This rural–urban obesity disparity is especially pronounced among young adults aged 20–39, with rural residents experiencing a 54% higher obesity rate (Rate Ratio [RR], 1.54; 95% CI, 1.34–1.77) [10].

CVD risk factors such as obesity and physical inactivity are exacerbated by environmental barriers resulting from limited access to recreational spaces and healthy food [11]. While proximity to agriculture may suggest higher vegetable consumption, this assumption does not reflect the broader rural U.S. reality. In many rural areas, restricted access to affordable, nutritious foods and higher rates of food insecurity negate any potential dietary benefits of living near farmland [12, 13].

Contrary to popular belief, rural residents are not more physically active than their urban counterparts. National trends indicate that physically demanding labor has declined in rural areas, and leisure-time physical activity remains lower compared to urban settings [10]. Multivariate analyses demonstrate that rural residence is independently associated with higher odds of obesity (OR, 1.36; *P* < 0.001). This disparity is partly attributable to lower educational attainment, reduced neighborhood income, and less supportive built environments characterized by limited access to parks, fitness centers, and full-service restaurants [14].

Subsequently, rural populations have higher rates of coronary heart disease (6.7% vs. 4.3%) [10] and consistently exhibit higher CVD mortality rates compared to urban counties [15, 16]. Rural populations also face significant healthcare access challenges due to transportation barriers, workforce shortages, and the growing issue of"cardiology deserts,” defined as a county with limited or no access to heart specialists, often requiring long commutes for care [17]. One in three underrepresented ethnic minorities live in counties described as cardiology deserts [17]. Access to cardiovascular care in rural areas is hindered by multiple structural barriers, including hospital closures and inadequate broadband infrastructure, which limit both in-person and telehealth services. Although the expansion of telemedicine during the COVID-19 pandemic improved outreach to rural populations distant from specialty clinics, significant disparities persisted. Notably, Black patients and older adults remained less likely to access specialty cardiovascular care via telehealth [18], highlighting the need for policy interventions that address not only geographic isolation but also sociodemographic inequities.

### Health Inequity as a Function of Sex and Gender

Over 60 million women in the U.S. live with CVD. Women are more likely to die from a myocardial infarction or develop heart failure post-discharge compared to men [19]. These disparities are further compounded during reproductive years, where CVD accounts for 13% of maternal deaths. Postpartum follow-up is often inadequate, especially for those with high-risk pregnancies [19]. Despite this, only 22% of physicians and 42% of cardiologists feel well-prepared to assess CVD in women, reflecting critical gaps in training and awareness [19]. Addressing these disparities could add 1.6 million life years and yield $28 billion in economic gains annually by 2040 [19]. Women remain significantly underrepresented in cardiovascular clinical trials, despite cardiovascular disease being the leading cause of death among women [20–22]. As a result, clinical guidelines and drug dosing recommendations are often based on data predominantly from men, overlooking important sex-specific differences in disease presentation, drug metabolism, and treatment response.

Transgender and nonbinary (TNB) individuals face significant cardiovascular health challenges, which are compounded by a lack of tailored research and inconclusive evidence on the effects of gender-affirming hormone therapy (GAHT) [23]. Minority stress, stigma, and limited access to affirming care further exacerbate cardiovascular risks in this population [23]. Studies have shown that transgender women have a higher odds of diabetes (aOR 1.45, 95% CI 1.05–1.99), stroke (aOR 1.88, 95% CI 1.16–3.03), coronary heart disease (aOR 1.90, 95% CI 1.34–2.68), and myocardial infarction (MI) (aOR 2.98, 95% CI 2.14–4.17) compared to cisgender men [24, 25].

## Health Inequity as a Function of Income and Education

Differences in CVD health outcomes have been observed when stratified by income. Individuals in the top 20% income bracket with a college degree have been observed to have the lowest prevalence of CVD conditions: congestive heart failure (CHF) (0.5%), angina (1.4%), MI (1.7%), and stroke (1.1%) [25]. In contrast, individuals in the bottom 80% income bracket without a college degree experience substantially higher rates CHF (3.0%), angina (2.8%), MI (3.9%), and stroke (3.4%) [25]. Compared to the top-income, college-educated group, individuals in the bottom 80% without a college degree had markedly elevated odds of CVD (aOR 2.36–6.52) [25]. Even among college graduates, those in lower income brackets experienced increased odds of disease (aOR 1.48–3.67) [25]. Notably, non-college graduates in the top income group also exhibited elevated odds for CHF and MI, highlighting the independent and compounding effects of both income and education on cardiovascular risk [25].

## Health Inequity as a Function of Race, Ethnicity, Heritage and Geography

Although race is a social construct based on perceived physical characteristics, it continues to shape health outcomes due to its association with structural and social determinants of health [26]. The *All of Us* cohort showed a higher adjusted odds of CVD among Black individuals (aOR 1.21; 95% CI, 1.16–1.27), particularly among those with lower income, disability, or advanced age [27]. By 2060, an estimated 59.9% of Black adults will have hypertension and 35.9% dyslipidemia, markedly higher than those projected for White populations, whose CVD burden is expected to decline [28]. Among American Indians and Alaska Natives, cardiovascular disease is the leading cause of death with mortality rates 20–50% higher than in White Americans [29]. The burden is especially stark in younger age groups: 36% of CVD deaths among Native Americans occur before age 65, compared to only 14.7% for white patients [29]. Although Asian American populations exhibit lower overall CVD risk, subgroup analyses reveal significant heterogeneity with Indian Americans having the highest ischemic heart disease mortality, and Vietnamese Americans having the highest cerebrovascular mortality amongst Asian subgroups [30]. These disparities are compounded by persistent underrepresentation in research; less than 0.17% of NIH funding between 1992 and 2018 was allocated to Asian American health studies [30].

## Health Inequity as a Function of Genetic Ancestry

To address cardiovascular inequities more effectively, we must move beyond race as a proxy and focus instead on genetic ancestry, which more precisely reflects biological variation and its interaction with the social determinants of health. Ancestry-specific genetic variants contribute to differences in disease susceptibility, treatment response, and access to precision therapies. For example, variants linked to hypertension and diabetes are more prevalent in individuals of African and South Asian ancestry, increasing their baseline CVD risk [31]. Pharmacogenomic differences also influence drug efficacy and safety; when assessed in aggregate, the SNP rs1937506 lowers blood pressure in Europeans, raises it in Hispanics, and has no effect in Africans [32]. In African Americans, higher European ancestry is associated with a lower risk of peripheral artery disease [33, 34] However, actionable variants for conditions such as hypertrophic cardiomyopathy are significantly less common in populations with African-ancestry, which limits access to targeted therapies [35].

The mutated variant of Transthyretin exemplifies how overlooking ancestry can perpetuate disparities. The Valine to isoleucine mutation at position 122 (V122I) is the most common inherited mutation that may result in amyloid transthyretin cardiomyopathy (ATTR-CM) in the U.S. The mutation has been predominantly seen in individuals of western African descent and carries a genetic frequency of 3–4% in African Americans. This variant is a major contributor to ATTR-CM, a progressive and often fatal form of heart failure [36, 37]. Moreover, simply carrying the V122I mutation is associated with incident HF compared to noncarriers [37]. Despite its prevalence, ATTR-CM remains underdiagnosed in Black communities, leading to missed opportunities for early intervention, and many may not be aware of their genetic risk [36, 37].

## Health Inequity as a Function of Immigration

Health inequity is closely linked to immigration status, as immigrants often face unique barriers that contribute to disparities in health outcomes. Migrant populations in North America generally have a higher risk of CVD compared to host populations. These disparities are often driven by social determinants such as poverty, food and housing insecurity, limited access to healthcare, and language or cultural barriers [38, 39]. The CVD risk profile amongst immigrant populations is not uniform. Hispanic/Latino immigrants may initially have lower cardiovascular risk, however over time this advantage often diminishes with longer residence in the host country [40, 41]. For others, such as South Asian immigrants_, the risk of CVD and metabolic diseases is higher than in native-born populations [40, 42]. Additionally, the effectiveness of standard cardiovascular risk markers and treatments may differ in immigrant populations, further complicating care and perpetuating inequities [43].

## Section III: The Dynamic Evolution of Understanding the Root Cause

### The 3 Eras of Social Determinants of Health Research

**Fig. 1:**
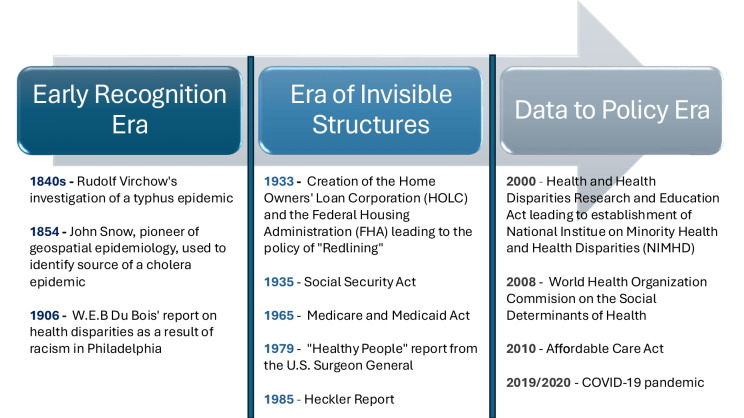
Eras of social determinants of health research

Although the concept of social determinants of health has sparked considerable interest over the past 20 years, it has been recognized both directly and indirectly as the root cause of health inequities for centuries. Our operationalization of the concept has evolved and can be reflected in three major eras across history: *The Early Recognition Era (19th to early twentieth century), The Era of Invisible Structures (Mid-20th Century), and The Data-to-Policy Era (21st Century) (*Fig. 1*)*.

#### The Early Recognition Era

The roots of the social determinants of health can be traced back to 19th-century thought leaders and researchers during widespread public health crises in the *Early Recognition Era*. Rudolf Virchow, a German pathologist and politician, is credited with being one of the earliest researchers to elucidate this connection in the 1840s. He concluded that poor housing, unemployment, and low education were the foundation of the worse Typhus epidemic outcomes in poverty-stricken Upper Silesia [44]. His conclusions were the first to connect social policy and health outcomes, introducing a concept of social medicine. Not long after, John Snow pioneered geospatial epidemiology by identifying the Broad Street pump as the source of the 1854 cholera epidemic in London, demonstrating the utility of geospatial mapping to track disease and the predisposing factors of poverty that affect disease transmission [45]. This era would end with the highly underrecognized but critically important revelations by W.E.B Du Bois in his study *The Philadelphia Negro* and report “The Health and Physique of the Negro American” which confronted the predominant belief of racial inferiority of African Americans and outlined how racism drove worse economic conditions, housing, education, forms of employment, and access to care resulting in health disparities [46, 47]. Du Bois mapped the clusters of poverty in the Seventh Ward of Philadelphia and demonstrated their relationship to race and health, becoming one of the unsung pioneers in social epidemiology [46]. Although the recognition of the social drivers of health was developed in this era, it was slow to enter into policy worldwide [48].

#### The Era of Invisible Structures

Exiting the First World War, the United States saw a booming economy through the 1920 s as the Great Depression led to unprecedented rates of unemployment and poverty in 1929. As a result, the country began to shift its focus to social welfare and health through the enactment of the Social Security Act in 1935 and decades later the Medicare and Medicaid Act in 1965 [49]. This would mark the beginning of *the Era of Invisible Structures*, a period during which systemic forces subtly but powerfully modified the social drivers of health to benefit US citizens, but not all equally. At the same time, there was limited public discourse and progress in the research of such social drivers. The goal of the Social Security Act was to generate economic provisions for Americans. The policy excluded agricultural and domestic workers, disproportionately limiting its benefits to groups more likely to hold these roles at that time such as African Americans [49]. This would only be compounded by the development of the Home Owners’ Loan Corporation (HOLC) and the Federal Housing Administration (FHA) as part of the New Deal in 1933, aimed at stimulating the housing market. In a belief that “incompatible racial groups should not be permitted to live in the same communities” as stated in their Underwriting Manual, the FHA would explicitly deny African-American mortgages in White neighborhoods, under-appraise African American homes, and refuse to insure mortgages in predominantly African-American neighborhoods in a practice known as “redlining” [50]. These policies not only resulted in wealth disparities but also exacerbated health disparities that have persisted to the current day along those historical red lines. For example, Black patients currently living in zip codes with higher redlining proportion are more likely to develop heart failure (HR, 1.08; 95% CI, 1.04–1.12, *p* < 0.001), have a higher risk of MI (HR, 1.148; 95% CI, 1.011–1.303; *P* < 0.00), and have a higher risk of death from all causes (HR, 1.129; 95% CI, 1.072–1.190; *P* < 0.001) [50, 51]. At the tail end of this era, we begin to see greater attention to social drivers of health as reflected in the landmark “Healthy People” report from the Surgeon General in 1979. However there remained little attention to differences in social determinants of health amongst specific demographic groups [52]. This would begin to slowly change with the 1985 Heckler Report which mobilized the US Department of Health and Human Services to begin addressing health care disparities and transitioned the field into the third era at the beginning of the twenty-first century [1].

#### The Data to Policy Era

The third era in the evolution of the social determinants of health is *the Data to Policy Era*, which captures the rapid increase in research and focus on new policies aimed at addressing the social determinants of health. The beginning of this era can be marked by the Health and Health Disparities Research and Education Act passed in 2000, leading to the establishment of the National Institute on Minority Health and Health Disparities (NIMHD) to oversee the national health disparities agenda for the nation [53]. Out of this policy also came the landmark report from the Institute of Medicine, *"Unequal Treatment,*"which explicitly outlined systemic racism as a driver of health disparities and called attention to multilevel approaches to address them [54]. Across the world, researchers engaged in deep analysis to better characterize health disparities throughout this era and the research efforts culminated with the World Health Organization Commission on the Social Determinants of Health which developed a conceptual framework for the social determinants and a global agenda to tackle their root causes [55–59]. Shortly after, the United States released the Healthy People 2020 report, which outlined the nation’s goal to achieve health equity and introduced 5 social determinant domains commonly used in disparities research [60]. These efforts ultimately contributed to the development of the Affordable Care Act, which increased access to healthcare and prevention to low-income individuals along with data collection mandates to enhance health disparities monitoring across demographics [61]. The end of this era is marked by the 2019 COVID-19 pandemic, whose outcomes underscored the global impact of social determinants of health. In contrast to *the Early Recognition Era*, the contemporary features of comprehensive data collection to inform policy and address health inequities are a defining characteristic of *the Data to Policy Era's* response to the COVID-19 pandemic, shaping the current landscape of social determinants of health [62]. The evolution of this era will be defined through innovative multisector partnership to codify effective policy across local, state, national, and international levels which was modeled in the response to the COVID-19 pandemic to be tangible when intentionally prioritized.

### Biological Effects of the SDOH

A growing body of literature is exploring the biological mechanisms by which SDOH contribute to health inequities. Central frameworks include the chronic stress and allostatic load model of impact where prolonged exposure to social stressors activates and adversely maintains the body’s stress response leading to functional decline over time [63–65]; cardiovascular and metabolic dysregulation particularly with the recently characterized cardio-kidney-metabolic syndrome (CKM) [66–68]; epigenetic modifications such DNA methylation [69–71]; and adverse cellular aging such as telomere shortening [72–74]. The genetic and epigenetic models have created avenues for precision risk prediction with the social determinants of health. In this section, we will highlight the key mechanistic models that explain the relationship between social determinants of health and cardiovascular disease.

#### Chronic Stress and Allostatic Load

One of the first and widely supported biological mechanisms connecting SDOH and health inequities is the chronic stress and allostatic load framework. Allostatic load represents the cumulative burden imposed on the body as a result of chronic stress and is commonly indexed using biomarkers from the immune, metabolic, and cardiovascular systems [63]. There have been multiple studies that have linked a higher allostatic load with worse cardiovascular outcomes, including heart failure and cardiovascular mortality [75, 76]. In addition, Black adults have been found to have a higher allostatic load than White adults, and foreign-born adults have lower allostatic load than U.S.-born adults, reflecting the impacts of various psychological stressors such as discrimination and racism [64, 65]. A systematic review of 22 studies examining the relationship between allostatic load and CVD found that a higher allostatic load is associated with a higher risk of CVD; however, the association was stronger with the baseline value, and fewer significant relationships were observed over longitudinal changes [63]. This suggests that allostatic load may be most effective in cross-sectional analyses and has limited utility in tracking changes over time. This also makes it impossible to determine whether higher allostatic load causes disease or is a consequence of it. In addition to this limitation, there is also no standardized calculation for allostatic load, resulting in the problematic interpretation of results across studies [63]. While allostatic load offers a valuable measure of chronic stress patients face, its limitations make it difficult to interpret on its own and limit its utility.

#### Cardiovascular and Metabolic Dysregulation

Another key biological pathway linking SDOH and adverse cardiovascular outcomes is the cardiovascular and metabolic dysregulation framework, now formally conceptualized as cardio-kidney-metabolic syndrome (CKM). As defined by the American Heart Association, CKM was introduced to capture the interconnected role of the disease states, the shared origin, and consequently, overlapping treatments. CKM is defined as any presence of atherosclerotic cardiovascular disease, heart failure, chronic kidney disease, diabetes, obesity, and metabolic syndrome [68]. CKM has been shown to disproportionately affect individuals living in areas of higher deprivation with CKM-related age-adjusted mortality significantly higher in the most socially deprived counties (572 vs. 454 per 100,000; *p* < 0.001) and in non-metropolitan compared to metropolitan areas (521 vs. 476 per 100,000) [67]. Another study found that communities facing higher rates of food insecurity and poorer air quality have higher rates of CKM mortality [66]. These findings, along with the holistic profiling that CKM enables, may be beneficial for population health-level risk stratification. Although a relatively young concept, further research is needed to characterize better the grading of CKM, its thresholds, and the mediating relationship with SDOH.

#### Genetics and Epigenetics

The AHA has emphasized the growing recognition of genetics and genomics on the development of CVD and the role SDOH in modulating genetic risk and health disparities [77, 78]. There have been over 250 loci linked to CAD identified through genome-wide association studies (GWAS), with more being discovered using multi-ancestry GWAS uncovering novel loci in diverse populations [79, 80]. However, diversity in genetics research is still insignificantly replete, often limiting generalizability to those of European descent. Emerging studies continue to explore new methods for diversifying repositories to enhance risk prediction and gain a deeper understanding of genomic variance within and between groups [79–82].

Monogenic or single-gene mutations have been found across GWAS to be associated with heart failure, CHD [79, 82, 83]. However, the ability of single-gene variants to explain community-level variability in disease is limited due to their rarity. In contrast, polygenic risk scores (PRS) enhance predictive ability by integrating the cumulative effect of multiple genetic variants [82, 84]. Research has shown the potential of PRS to improve risk stratification for CAD, HLD, type 2 diabetes, and atrial fibrillation, with enhanced detection when greater diversity is incorporated into the GWAS [82, 84]. Although there have been polymorphic genetic variants identified that put people at heightened risk for cardiovascular diseases, they have been found to account for only a fraction of the burden of disease, pointing towards a need to understand how social and environmental determinants augment and/or modify cardiovascular diseases (CVD) risk [82].

Epigenetic mechanisms, particularly telomere shortening and DNA methylation, have been shown to provide insight into how SDOH can influence cellular function and increase CVD risk. Of the two, DNA methylation has the most robust data on the relationship with SDOH. Telomere shortening is a biomarker of cellular aging that occurs naturally over time. It is associated with chronic age-related conditions and has been shown to be sensitive to chronic stress and adverse social conditions [72]. Studies examining the relationship between telomere length and CVD show an inverse relationship between telomere length and CHD and HF recovery [85–87]. Telomere length is not a specific indicator and many factors influence its length making it difficult to isolate the impact of social factors on cardiovascular risk [72, 73, 88].

In contrast to telomere length, DNA methylation has been shown to have a stronger association with CVD [89, 90]. DNA methylation may provide a more robust measure of the biological impact of SDOH. Accelerated biological aging can be identified granularly through the addition of methyl groups in CpG dinucleotides. The patterns of methylation are then associated with disease or mortality and can be measured over time to form a biological “clock”. Multiple epigenetic “clocks” have been developed, including GrimAge, PhenoAge, DunedinPoAm, and DunedinPACE, which have demonstrated significant associations with morbidity, mortality, and the incidence of CVD [71, 88, 91–93]. Analysis of these clocks have also indicated that neighborhood-level stressors, such as living below the poverty line accelerate aging [70].

In the study by Shen et al., African Americans who lived above the poverty line still experienced a faster rate of aging, reflecting the importance and relevance of non-economic determinants and stressors that accelerate aging, such as discrimination, class inequality, housing, among others (69). These new measures can help us identify deleterious changes before chronic diseases are established and thus help measure the short- and medium-term impacts of interventions aimed at addressing health inequities [93, 94]. Although these epigenetic clocks are promising, more robust and diverse data are needed to validate the measures [95, 96].

#### Future Precision Cardiovascular Risk Stratification

Genetic factors alone account for only a fraction of the diseased phenotype. A more informative and individualized risk profile can be developed when considering genetic factors, environmental exposures, and epigenetic modifications from exposure to social determinants of health. In a UK biobanking study examining the chronic exposure of air pollutants on the risk of MI in patients with variable genetic risk, results demonstrated that participants with high genetic risk for MI and high air pollutant exposure had ∼250% (PM2.5), 324% (PM10), 286% (NO2), and 293% (NOx) higher MI risk compared with those at low genetic risk and exposed to low concentrations of air pollutants—demonstrating a multiplicative interaction that was greater than either exposure alone [97, 98]. Genetic factors may play a crucial role in how social determinants of health influence the risk of CVD, as a person’s genetics and epigenetics may have variable effects on phenotypic expression depending on exposure. Precision medicine is the next generation of risk stratification.

### Capturing the Social Determinants: Now and the Future

#### Individual Factors to Indices

Initially, social determinants of health were identified through screening forms to assess patient-level risk factors. This phenomenon was first observed as early as the 1930 s, when the Census Bureau and the National Tuberculosis Association stratified morbidity data by socioeconomic status [95]. The evolution to capturing population-level data has allowed for better analysis of associations across social determinants of health domains. Through time, our understanding of the interconnected nature of SDOH led to an interest in developing indices to capture a single snapshot of the state of health for communities. The 1972 article “Toward a Social Indicator of Health” first documented the framework needed to capture “social indicators” of health, drawing on learned experiences from already established economic indicators developed to reflect the growth or decline of the economy [99]. This was followed shortly after by the first multivariate analysis in 1978, which indexed each county in Mississippi using 55 indicators of health grouped into the following categories: resource population, economics, geriatrics, social problems, preventive medicine, nutrition, mortality, and disability [100]. In this very early attempt to index counties relative to each other, they found that the highest ranking counties with “good health” (defined as countries that had a higher percentage of positive health factors across factors) were all grouped in the southern portion of the state near the water and the counties with the “worst health” were all located deep inland—highlighting very early a geographical divide on health and health outcomes [100]. This has evolved over the years to the early 2010 s, when the first contemporary index score to capture social determinants of health was developed. See Appendix 1 for review of current and future SDOH indices relevant to cardiovascular outcomes. 

#### Next Generation Index for Cardiovascular Disease

**Fig. 2:**
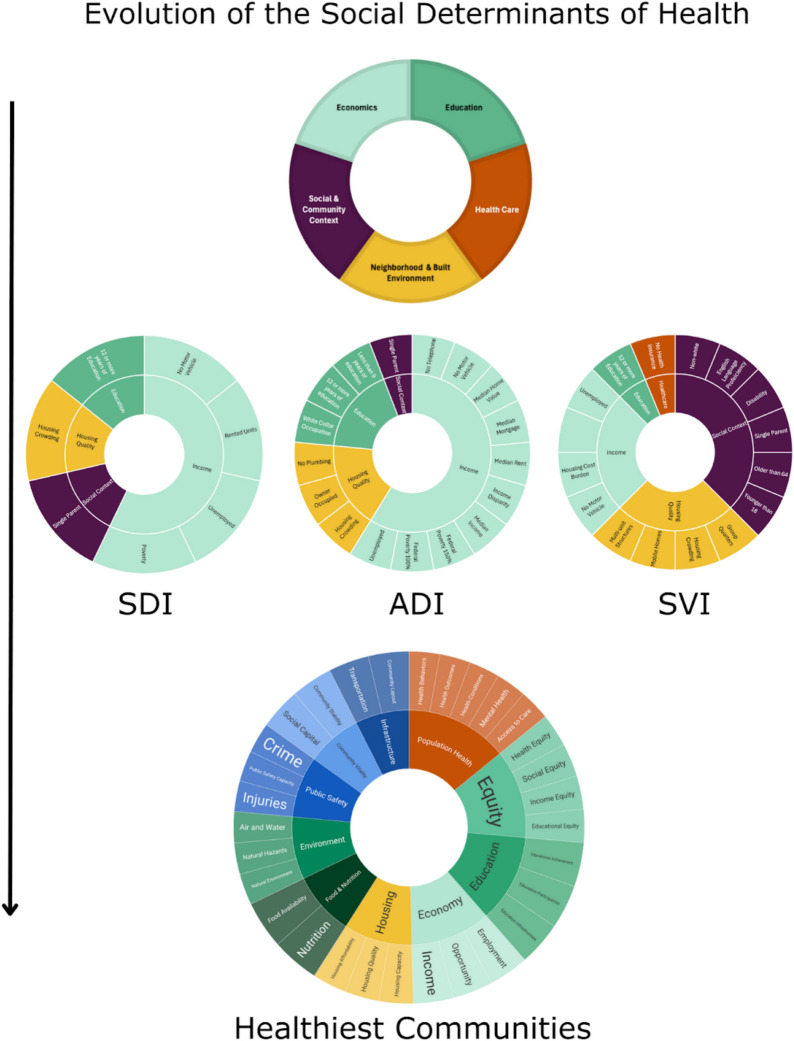
Represents the transition from standard individual categories to more complex indices capturing more dimensions of the social determinants of health. It can be visualized the overrepresentation of income in the most utilized indices of this era (ADI, SDI, and SVI). But as the Healthiest Communities framework is assessed, the multidimensional and diverse range of factors is appreciated and can be utilized to make a cardiovascular-specific index. The “Healthiest Communities” graphic was obtained and used with granted permission from US News and World Report (https://www.usnews.com/news/healthiest-communities/articles/methodology)

On the horizon of the SDOH indices is generating an integration of area level deprivation into practice. Once there is consensus around a tool for cardiovascular outcomes, clinicians need guidance on how to interpret and incorporate them into decision making. There needs to be an understanding of how traditional individual risk assessment intersects with a tailored population level risk assessment, as this combination has been shown to be most effective in predicting outcomes. This next generation deprivation index should also incorporate a more comprehensive range of social drivers than the current indices. The current model of SDOH has 5 domains and most of the indices utilize 4 of them. An example of a comprehensive model is the Healthiest Communities Index created by US News and World Report [101]. Although not studied as rigorously or validated, the framework takes a comprehensive approach in naming 10 domains and 30 subdomains to define and rank 500 cities across the US (Fig. 2). Future studies should examine their dimensions and weighting to advance our scientific integration of the SDOH in cardiovascular risk prediction.

## Conclusions

The journey toward cardiovascular health equity reflects a broader reckoning with the structural forces that shape health outcomes. In the past, glaring disparities in cardiovascular disease incidence, management, and mortality were tolerated as unfortunate byproducts of a broken system. These inequities were not only predictable; they were preventable. The present moment, however, is defined by urgency and opportunity. We now possess the data, the tools, and the collective awareness to confront injustice in cardiovascular care head-on. From race-conscious clinical guidelines to community-driven interventions, and from inclusive research practices to digital innovations that detect hidden risk, the field is steadily shifting from passive observation to active correction. See Appendix 2 for a review of current implementation solutions to advance cardiovascular health equity.

Yet the future demands more. True equity will not emerge from incremental change but from sustained disruption of outdated systems, biased algorithms, and unequal access. It will be built by a diverse workforce committed to justice, driven by predictive technologies that serve rather than exclude, and anchored in policies that prioritize prevention, access, and trust. Health equity in cardiovascular medicine is no longer a distant ideal, it is the defining mandate of modern care. The past has exposed the gaps. The present is aiming to close them. The future must eliminate them entirely and create a system where equity is at the core of every function.

## Key References

- Abdalla SM, Rosenberg SB, Maani N, Contreras CM, Yu S, Galea S. Income, education, and the clustering of risk in cardiovascular disease in the US, 1999–2018: an observational study. The Lancet Regional Health – Americas [Internet]. 2025 Apr 1 [cited 2025 Apr 25];44. Available from: https://www.thelancet.com/journals/lanam/article/PIIS2667-193X(25)00049-3/fulltext.
  - Findings from this study suggest that while higher-income groups saw significant reductions in CVD risk, the lowest-income group experienced no meaningful change.
- Faul JD, Kim JK, Levine ME, Thyagarajan B, Weir DR, Crimmins EM. Epigenetic-based age acceleration in a representative sample of older Americans: Associations with aging-related morbidity and mortality. Proc Natl Acad Sci U S A. 2023 Feb 28;120(9):e2215840120.
  - Findings from this study suggest that epigenetic age acceleration tools in conjunction with traditional social and behavioral measures of risk may enhance the predictability or morbidity and mortality outcomes later in life.

## Supplementary Information

Below is the link to the electronic supplementary material.

Supplementary file1 (DOCX 62 KB)

Supplementary file2 (DOCX 21 KB)

## Data Availability

No datasets were generated or analysed during the current study.

## Acronyms

ADI: Area Deprivation Index
ASCVD: Atherosclerotic Cardiovascular Disease
ATTR-CM: TTR Amyloid Cardiomyopathy
CAD: Coronary Artery Disease
CAD: Coronary Heart Disease
CHF: Congestive Heart Failure
CKM: Cardio-Kidney-Metabolic Syndrome
CMS: Centers for Medicare & Medicaid Services
CVD: Cardiovascular Disease
EBP: Evidence Based Practices
FHA: Federal Housing Administration
GWAS: Genome Wide Association Studies
HF: Heart Failure
HLD: Hyperlipidemia
HOLC: Home Owners’ Loan Corporation
HRSA: Health Resources and Services Administration
MI: Myocardial Infarction
SDI: Social Deprivation Index
SDOH: Social Determinants of Health
SVI: Social Vulnerability Index
U.S.: United States
