# Supplementary material for: Echoes of Inequity: A Critical Examination of the Past, Present, and Future of Cardiac Health Equity
Source: Curr Cardiol Rep. 2025 Sep 19;27(1):134. doi: 10.1007/s11886-025-02275-y (PMC12449390; doi:10.1007/s11886-025-02275-y)
Supplement: Supplementary file 1 — Supplementary file1 (DOCX 62 KB) [file 11886_2025_2275_MOESM1_ESM.docx]

**Appendices:**

**Appendix 1**

***Area Deprivation Index***

There are multiple health indices used to capture an aggregate of social risk to determine a particular outcome. Supplementary Table 1 outlines 3 of the major indices utilized in current research with the most robust data comparing their origin and function, ultimately determining their utility. Although there have been other area-based deprivation indices used in Europe and Australia, this review will focus on those developed in the United States(102,103). The first contemporary community level index that was developed is the Area Deprivation Index (ADI) which was developed by a Health Resources and Services Administration (HRSA) senior researcher, Gopal Singh in 2003. This index was developed to provide a composite area-based score to understand how community level socioeconomic disadvantage impacts changes in all-cause mortality over time(104). Through principal component analysis, Singh identified 17 measures that had a strong factor loading mortality which were included as their relative weighted fraction in the index based on their factor loading (104). ADI was further validated, modified, and made publicly available in the form of national rankings (1-100) and state level rankings (1-10) by a research team at the Center for Health Disparities Research at the University of Wisconsin School of Medicine and Public Health in 2018 (96,105). Since publicly available, there has been a dense amount of research examining the association of ADI and a range of cardiovascular outcomes, along with utilization in policy development and implementation (98,106,107) . From a policy standpoint, examples of policy use cases include the Centers for Medicare & Medicaid Services (CMS) Accountable Care Organization Realizing Equity, Access, and Community Health (ACO REACH) model that increases payments to facilities that care for patients in areas of high deprivation recognizing the high degree of disease burden and barriers to care(108,109). A systematic review of literature using ADI since 2015 found 222 original research articles utilizing ADI or the Neighborhood Atlas (online version of ADI) to examine associations with health outcomes with 17% focusing on cardiovascular health (98). Studies have identified that increased ADI scores are associated with increased 30 day cardiovascular mortality, heart failure, ischemic heart disease, and cardiac arrhythmias(98,110) .

***Social Vulnerability Index***

The Social Vulnerability Index (SVI) developed by the Centers for Disease Control and Prevention following the Pandemic and All-Hazards Preparedness Act signed by President George W. Bush in 2006, aimed to help public health officials allocate resources and plan interventions in communities most at risk during natural disasters(111,112). The SVI is composed of 16 social factors grouped into 4 themes: socioeconomic status, household composition and disability, minority status and language, and housing type and transportation (Supplementary Table 1) (111). SVI has been instrumental in guiding public health response to emergencies. During the COVID-19 pandemic, research interest grew in analyzing how SVI was associated with COVID-19 related cardiovascular outcomes. Later, the SVI was used among researchers to understand general cardiovascular disease risk across socially vulnerable populations(16,107,113–115). Supplementary Table 1 outlines some of the observed associations between SVI and cardiovascular outcomes of which include CHD prevalence and risk of CVD mortality (16,107). Additionally, the American College of Cardiology has integrated SVI into their Health Equity Heat Map to help institutions and clinicians to better tailor interventions for the communities around them (54).

***Social Deprivation Index***

Developed in 2013, the Social Deprivation Index (SDI) is the next largest used index. SDI was initially developed by Butler et al as a part of the Robert Graham Center utilizing 2009 American Community Survey Data and has been adapted to be a composite measure of 7 demographic characteristics to quantify the socioeconomic variation in health outcomes (Supplementary Table 1) (116,117) . The index was formulated utilizing a similar method of factor analysis as demonstrated with previously described indices above (116). Each factor was weighted based on its factor loading score, ultimately leading to a decile index. Researchers have explored the relationship between SDI and cardiovascular disease, although with less robust evidence than ADI. A greater SDI is associated with increased risk of heart failure and cardiovascular mortality (51,107,118) . Though SDI has not been used as widely in public policy, it has been recently integrated in the new American Heart Association Predicting Risk of CVD EVENTs (PREVENT) equations to provide a race-free, place-based cardiovascular disease predictive model (119) (53).

***Are Indices the Way to Go?***

Although the area-level indices are associated with adverse cardiac morbidity and mortality, there is still considerable room for improvement in their utility. First, it is essential to understand the primary purpose of the index tool. SVI was developed to direct public health resources during an emergency, not for predicting cardiovascular outcomes. Similarly, ADI and SDI were created to measure the relationship between socioeconomic burden across regions and mortality from all causes, not just cardiac causes. When strategizing an index to capture the impact of SDOH on cardiovascular morbidity and mortality, it needs to be specifically modeled to determine an association with the outcomes of interest, such as cardiovascular mortality. Although many of the indices have been found to have associations with cardiovascular mortality, we can significantly enhance the discrimination between different levels of social deprivation through this. In addition to developing indices that are tailored for cardiovascular outcomes, the index variables must have a meaningful impact on the outcomes of interest. The current variables in ADI, SDI, and SVI have a varying level of influence on the association with cardiovascular disease. SES has been shown in multiple studies to account for the variability in CVD risk between areas of high and low levels of deprivation (114,120). This may be reflective of SES capturing multiple social factors that influence health outcomes, such as access to care, insurance, and health literacy. It will be essential to understand that a fraction of the variables may explain the difference between the levels of deprivation. This also contributes to the critical consideration of geographically weighing respective variables.

Currently, all three indices have internal weights based on their factor loading value when they were created. But how does regional variation determine the impact of each variable in the model? Is the ownership of a motor vehicle as crucial in New York as it is in Detroit? Does the cost of housing in Sacramento have the same effect as Durham? Lastly, the lack of standardization in variables and overlap in certain categories can lead to overemphasis in specific categories or “double counting” (121,122). For example, in the ADI index, there are variables for “median home value” and “median gross rent” which may be capturing the same dimension of social deprivation but are counted twice. Despite the data collected over the past decade, there is still room to improve the utilization of SDOH indices in predicting cardiovascular outcomes.

**Appendix 2**

**Section IV: Implementing Solutions**

**Identifying the Gap: Equity, Low- and High-Value Care**

A foundational step in addressing disparities is identifying equity gaps and evaluating the effectiveness of existing interventions across diverse populations. As discussed earlier under *Health Inequities*, understanding these disparities involves not only tracking outcomes by race, income, and education but also evaluating how and where care delivery fails to serve all groups equitably.

This includes recognizing when widely used practices are ineffective or harmful in specific populations, as well as identifying where high-value practices remain underutilized among historically marginalized groups.

**Selecting and Adapting Evidence-Based Practices (EBPs) to Address Disparities**

Many evidence-based practices (EBPs) for CVD prevention and management were not designed or tested with underrepresented populations in mind. As such, adaptation is essential. Moise et al emphasized that selecting and tailoring EBPs must involve addressing structural barriers, fostering community engagement, and mitigating power imbalances to ensure cultural relevance and impact (123).

Implementation science offers frameworks to operationalize this process. For instance, community-based models such as hypertension management programs in barbershops and churches have demonstrated striking improvements in blood pressure control (68% vs. 11% with standard care) (124). These successes underscore the importance of tailoring interventions to local context and culture.

Simultaneously, de-implementation of low-value care is critical to advancing equity. Singh et al analyzed over 450 studies and 186 unique quality improvement (QI) interventions targeting CVD. While patient support programs used in 68.2% of interventions had mixed effects on clinical outcomes, they consistently improved education and satisfaction (125).

Community-based programs among other strategies show promise in reducing major adverse cardiovascular events (MACE)(123). Technology-enabled interventions, including telehealth, digital monitoring, and wearables have been shown to reduced hospitalizations and improved cholesterol and medication adherence. However, results for blood pressure control were inconsistent (123). High-intensity provider training emerged as a practical approach to enhance clinical guideline adherence, emphasizing the role of workforce education in equitable implementation (123)

**Embedding Equity: Structural, Systemic, and Community-Level Solutions**

Achieving sustainable change in cardiovascular health requires confronting the structural and systemic drivers of inequity. This begins with diversifying the cardiology workforce to improve representation and foster cultural concordance between providers and patients. Integrating SDOH into routine clinical care is crucial for addressing the upstream causes of CVD, including housing instability, food insecurity, and environmental exposures. Expanding culturally tailored, community-based programs can extend the reach and effectiveness of high-value interventions, especially in underserved populations. Ultimately, policy reforms and increased investment in preventive care are crucial to closing persistent gaps in healthcare coverage and access, thereby ensuring that equitable cardiovascular care becomes a reality for all.

Clinical trial diversity remains a foundational issue to be addressed in order to ensure novel therapies benefit all populations. Equity must also be woven into professional education. Embedding training on cultural competence, implicit bias, and structural determinants of health within medical curricula is essential to shifting practice norms and reducing disparities (126).

Equitable implementation must extend beyond traditional clinical settings. Innovative strategies such as health fairs, mobile clinics, and local partnerships help anchor care in community needs rather than academic or institutional priorities. Accurate equity requires more than implementation; it demands co-creation with those most affected by disparities.

**Addressing Barriers to Implementing Evidence-Based Practices (EBPs)**
Historically marginalized populations frequently face structural barriers, such as food insecurity, unstable housing, and limited access to quality healthcare, that diminish the uptake and effectiveness of evidence-based cardiovascular interventions(127). Implementation science provides tools to mitigate these challenges by systematically incorporating input from key stakeholders, including patients, clinicians, community leaders, and policymakers. This inclusive approach ensures that context-specific barriers are identified and addressed at the outset. Frameworks such as the Consolidated Framework for Implementation Research (CFIR) and RE-AIM (Reach, Effectiveness, Adoption, Implementation, Maintenance) facilitate a structured evaluation of interventions, particularly across diverse populations and healthcare settings (128,129). As described by Moise et al, implementation challenges vary depending on the intervention level, whether community-based, healthcare system-wide, or policy-oriented, and often mirror deeper social determinants of health (123). By centering stakeholder engagement, implementation frameworks ensure that strategies are both equitable and contextually grounded, particularly for populations that have been historically excluded from traditional health interventions.

**Selecting and Deploying High-Value Cardiovascular Implementation Strategies**
Improving cardiovascular outcomes and closing disparity gaps requires the adoption of implementation strategies that are not only evidence-based but also culturally and structurally responsive to the populations they aim to serve. Among high-value approaches, population-based dashboards and risk-stratification tools, such as the PREVENT model, stand out as particularly effective. PREVENT offers a race-exempt yet race-conscious framework by incorporating socioeconomic status and geographic location into ASCVD risk prediction (130). Preliminary data suggest that these tools improve statin initiation and adherence, while also contributing to reductions in low-density lipoprotein (LDL) cholesterol levels.

Embedding community health workers (CHWs) into clinical care teams represents another impactful strategy. CHWs have demonstrated success in addressing social determinants of health, increasing patient engagement, and enhancing continuity of care, particularly among marginalized populations (131). This model has been effectively scaled in systems such as New Mexico’s Medicaid program. Finally, multicomponent interventions combining provider training, team-based care, patient education, and case management consistently produce the most durable improvements in outcomes. To advance equity meaningfully, these strategies must be intentionally tailored to reflect the cultural norms, lived experiences, and structural challenges of the communities they aim to benefit.

**Selecting and De-Implementing Low-Value Cardiovascular Strategies**
While the adoption of high-value interventions is essential, reducing the use of low-value or harmful practices is equally critical to achieving health equity. Nearly half of U.S. patients receive at least one low-value test or procedure each year, resulting in avoidable harms and financial burdens (132). For racial and ethnic minorities, this often manifests as a “double jeopardy”—facing both underuse of beneficial services and overuse of ineffective or unnecessary care (133). De-implementation science addresses this issue by identifying low-value practices, designing interventions to reduce their use, and assessing whether these changes are sustained over time. Research suggests that the manifestation of overuse can vary across subpopulations. The “double jeopardy” model highlights how marginalized groups may be disproportionately affected by both inappropriate and insufficient care.

In contrast, the “thermostat” model posits a uniform downscaling of care, both appropriate and inappropriate, for these groups (133). Without deliberate efforts to account for these dynamics, de-implementation strategies risk reinforcing disparities. Equity-oriented care, therefore, requires a dual commitment: expanding access to high-value interventions while actively dismantling the systems that perpetuate overuse of low-value care (134)

**Evaluating Implementation Success and Sustaining Interventions**
Ensuring the long-term impact of EBPs in cardiovascular care requires evaluation strategies that extend beyond individual clinical outcomes to include organizational, system-level, and policy-related effects. Implementation science frameworks emphasize the need to assess not only clinical efficacy but also implementation outcomes such as adoption, fidelity, sustainability, and cost-effectiveness.

Moise et al highlight key principles for evaluating implementation success, including adaptability, sustained stakeholder engagement, and the capacity to learn from both challenges and successes (123). Hybrid effectiveness-implementation designs—such as stepped-wedge trials—offer a rigorous yet flexible approach to simultaneously evaluating clinical and implementation outcomes in real-world contexts (135,136). These designs support iterative adaptation, which enhances both scalability and external validity. In dynamic healthcare environments, rigid adherence to initial protocols can hinder impact; instead, responsiveness to local context and flexibility are essential for driving meaningful change. Moreover, scalability should be considered from the outset to ensure equity-promoting interventions can expand without compromising cultural relevance or effectiveness (137).

Sustained impact also depends on embedding EBPs within existing healthcare infrastructures through mechanisms such as reimbursement reform, integration into quality metrics, or alignment with broader public health initiatives. Importantly, evaluators must examine whether implementation outcomes differ between historically marginalized and non-marginalized populations. Without this equity lens, even well-intentioned interventions may unintentionally perpetuate disparities.

**References**

102. Carstairs V, Morris R (1989) Deprivation: explaining differences in mortality between Scotland and England and Wales. BMJ 299:886–889

103. Phillips RL, Liaw W, Crampton P, Exeter DJ, Bazemore A, Vickery KD, Petterson S, Carrozza M (2016) How Other Countries Use Deprivation Indices—And Why The United States desperately needs one. Health Affairs 35:1991–1998

104. Singh GK (2003) Area Deprivation and widening inequalities in US mortality, 1969–1998. American Journal of Public Health 93:1137–1143

105. Kind AJH, Jencks S, Brock J, Yu M, Bartels C, Ehlenbach W, Greenberg C, Smith M (2014) Neighborhood socioeconomic disadvantage and 30-Day rehospitalization. Annals of Internal Medicine 161:765

106. Johnson AE, Zhu J, Garrard W, Thoma FW, Mulukutla S, Kershaw KN, Magnani JW (2021) Area deprivation Index and Cardiac Readmissions: Evaluating Risk‐Prediction in an Electronic Health Record. Journal of the American Heart Association. https://doi.org/10.1161/jaha.120.020466

107. Bevan GH, Nasir K, Rajagopalan S, Al-Kindi S (2022) Socioeconomic deprivation and premature cardiovascular mortality in the United States. Mayo Clinic Proceedings 97:1108–1113

108. (2021) Adjusting Medicare payments for social risk to better support social needs. Forefront Group. https://doi.org/10.1377/forefront.20210526.933567

109. (2024) Accountable Care Organization (ACO) Realizing Equity, Access, and Community Health (REACH) Model | CMS. https://www.cms.gov/newsroom/fact-sheets/accountable-care-organization-aco-realizing-equity-access-and-community-health-reach-model.

110. Berman AN, Biery DW, Ginder C, et al (2021) Association of socioeconomic disadvantage with long-term mortality after myocardial infarction. JAMA Cardiology 6:880

111. Flanagan, Gregory, Hallisey, Heitgerd, Lewis (2011) A social Vulnerability Index for disaster management. https://econpapers.repec.org/RePEc:bpj:johsem:v:8:y:2011:i:1:p:24:n:23. Accessed 24 May 2025

112. Pandemic and All Hazards Preparedness Act (PAHPA). In: https://aspr.hhs.gov/legal/pahpa/Pages/default.aspx. https://aspr.hhs.gov/legal/pahpa/Pages/default.aspx.

113. Islam SJ, Malla G, Yeh RW, et al (2022) County-Level Social Vulnerability is Associated With In-Hospital Death and Major Adverse Cardiovascular Events in Patients Hospitalized With COVID-19: An Analysis of the American Heart Association COVID-19 Cardiovascular Disease Registry. Circulation Cardiovascular Quality and Outcomes. https://doi.org/10.1161/circoutcomes.121.008612

114. Jain V, Rifai MA, Khan SU, et al (2022) Association between social vulnerability index and cardiovascular disease: A Behavioral Risk Factor Surveillance System study. Journal of the American Heart Association. https://doi.org/10.1161/jaha.121.024414

115. Terry K, Makhlouf M, Altarabsheh SE, Deo V, Petermann‐Rocha F, Elgudin Y, Nasir K, Rajagopalan S, Al‐Kindi S, Deo S (2023) Trends in cardiovascular disease mortality by County‐Level Social Vulnerability Index in the United States. Journal of the American Heart Association. https://doi.org/10.1161/jaha.123.030290

116. Social Deprivation Index (SDI). In: Robert Graham Center. https://www.graham-center.org/maps-data-tools/social-deprivation-index.html.

117. Butler DC, Petterson S, Phillips RL, Bazemore AW (2012) Measures of Social Deprivation That Predict Health Care Access and Need within a Rational Area of Primary Care Service Delivery. Health Services Research 48:539–559

118. Patel SA, Krasnow M, Long K, Shirey T, Dickert N, Morris AA (2020) Excess 30-Day heart failure readmissions and mortality in Black patients increases with neighborhood deprivation. Circulation Heart Failure. https://doi.org/10.1161/circheartfailure.120.007947

119. Khan SS, Matsushita K, Sang Y, et al (2023) Development and validation of the American Heart Association’s PREVENT equations. Circulation 149:430–449

120. Machado S, Sumarsono A, Vaduganathan M (2021) Midlife wealth mobility and long-term cardiovascular health. JAMA Cardiology 6:1152

121. Wehbe C, Baroud H (2024) Limitations and considerations of using composite indicators to measure vulnerability to natural hazards. Scientific Reports. https://doi.org/10.1038/s41598-024-68060-z

122. Petterson S (2023) Deciphering the Neighborhood Atlas Area Deprivation Index: the consequences of not standardizing. Health Affairs Scholar. https://doi.org/10.1093/haschl/qxad063

123. Moise N, Cené CW, Tabak RG, Young DR, Mills KT, Essien UR, Anderson CAM, Lopez-Jimenez F (2022) Leveraging implementation science for cardiovascular health equity: A scientific statement from the American Heart Association. Circulation. https://doi.org/10.1161/cir.0000000000001096

124. Victor RG, Lynch K, Li N, et al (2018) A Cluster-Randomized trial of Blood-Pressure reduction in Black barbershops. New England Journal of Medicine 378:1291–1301

125. Singh K, Bawa VS, Venkateshmurthy NS, et al (2021) Assessment of studies of quality improvement strategies to enhance outcomes in patients with cardiovascular disease. JAMA Network Open 4:e2113375

126. Douglass PL, Itchhaporia D, Bozkurt B, et al (2024) Achieving equitable cardiovascular care for all. JACC Advances 3:101050

127. Schwartz JK, Kringle EA, Burns SP, Hoyt CR, Harris KM, Tayeb S (2024) Cardiovascular Outcomes, Health-Promoting Behaviors, and Social Determinants: Structural Racism and the Behavioral Risk Factor Surveillance system. Health Equity 8:707–719

128. D’Lima D, Soukup T, Hull L (2022) Evaluating the Application of the RE-AIM Planning and Evaluation Framework: An updated Systematic review and exploration of Pragmatic application. Frontiers in Public Health. https://doi.org/10.3389/fpubh.2021.755738

129. Safaeinili N, Brown‐Johnson C, Shaw JG, Mahoney M, Winget M (2019) CFIR simplified: Pragmatic application of and adaptations to the Consolidated Framework for Implementation Research (CFIR) for evaluation of a patient‐centered care transformation within a learning health system. Learning Health Systems. https://doi.org/10.1002/lrh2.10201

130. Anderson TS, Wilson LM, Sussman JB (2024) Atherosclerotic cardiovascular Disease risk estimates using the predicting risk of cardiovascular disease Events equations. JAMA Internal Medicine 184:963

131. Knowles M, Crowley AP, Vasan A, Kangovi S (2023) Community Health Worker Integration with and Effectiveness in Health Care and Public Health in the United States. Annual Review of Public Health 44:363–381

132. Kini V, Breathett K, Groeneveld PW, Ho PM, Nallamothu BK, Peterson PN, Rush P, Wang TY, Zeitler EP, Borden WB (2022) Strategies to Reduce Low-Value Cardiovascular Care: A scientific statement from the American Heart Association. Circulation Cardiovascular Quality and Outcomes. https://doi.org/10.1161/hcq.0000000000000105

133. Helfrich CD, Hartmann CW, Parikh TJ, Au DH (2019) Promoting Health Equity through De-Implementation Research. Ethnicity & Disease 29:93–96

134. Pineles BL, Bonafide CP, Ashcraft LE (2024) Deimplementation of Ineffective and Harmful Medical Practices: A Data-Driven Commentary. American Journal of Epidemiology. https://doi.org/10.1093/aje/kwae285

135. Simon GE, Garner BR, Smith JD, et al (2025) Rollout trial designs in implementation research are often necessary and sometimes preferred. Implementation Science. https://doi.org/10.1186/s13012-025-01422-x

136. Hooper R (2021) Key concepts in clinical epidemiology: Stepped wedge trials. Journal of Clinical Epidemiology 137:159–162

137. Breathett K, Lewsey S, Brownell NK, et al (2024) Implementation Science to Achieve equity in heart Failure Care: A scientific statement from the American Heart Association. Circulation. https://doi.org/10.1161/cir.0000000000001231
