# Supplementary material for: Echoes of Inequity: A Critical Examination of the Past, Present, and Future of Cardiac Health Equity
Source: Curr Cardiol Rep. 2025 Sep 19;27(1):134. doi: 10.1007/s11886-025-02275-y (PMC12449390; doi:10.1007/s11886-025-02275-y)
Supplement: Supplementary file 2 — Supplementary file2 (DOCX 21 KB) [file 11886_2025_2275_MOESM2_ESM.docx]

Supplementary Table 1. Major geographic indices capturing the social determinants of health and their relationship with cardiovascular outcomes.

|  | Area Deprivation Index (ADI) | Social Vulnerability Index (SVI) | Social Deprivation Index (SDI) |
| --- | --- | --- | --- |
| Year | 2003 | 2011 | 2013 |
| Source | Health Resources Services Administration | Centers for Disease Control and Prevention | Robert Graham Center |
| Level of Analysis | Block Group | Census Tract | County, Census tract, Aggregated Zip Code Tabulation Area (ZCTA), and Primary Care Service Area (PCSA, v 3.1) |
| Original Purpose | Identify areas of high levels of socioeconomic disadvantage | Identify communities needing most support in disasters | Quantify levels of disadvantage across small areas and to evaluate their association with health outcomes and address health inequities |
| Domains | 17 Markers  -Education  -Income  -Employment  -Housing Quality | 16 Markers  -Socioeconomic status  -Household Characteristics  -Racial and Ethnic Minority status  -Housing Type and Transportation | 7 demographic characteristics from American Community Survey  -Percent living in poverty  -percent with less than 12 years of education  -percent single-parent household, percentage living in rented housing units  -percentage living in the overcrowded housing unit, percentage of households without a car, percentage unemployed under 65 |
| Cardiovascular Disease Associations | Associated with increased all cause and cardiovascular mortality after MI | Associated with worse rates of HTN, heart failure, stroke, and ischemic heart disease | Associated with heart failure and cardiovascular mortality |
| Effect Size (ꞵ-Coefficient, R^2^, AUC vs HR) | 30-Day Mortality Adjusted OR for group 1 vs group 4 valvular disease 1.63 (1.36-1.95), Heart Failure 1.29 (1.22-1.34), ischemic heart disease 1.34 (1.25-1.43), Cardiac Arrhythmia 1.41 (1.29-1.55) (69) | Rate ratio of CVD mortality of group 1 compared to group 4  1.84 [95% CI, 1.43–2.36] (7)  CHD Prevalence - R^2^=0.74 (84) | Premature CV mortality higher for higher SDI SDI when controlling for traditional CV risk factors (beta, 0.30; 95% CI, 0.26–0.34; P<.001; R2, 0.74) (71) |
| Site | <https://www.neighborhoodatlas.medicine.wisc.edu/> | [https://www​.atsdr.cdc​.gov/placeandhealth​/svi/data_documentation_download.html](https://www.atsdr.cdc.gov/placeandhealth/svi/data_documentation_download.html) | https://www.graham-center.org/maps-data-tools/social-deprivation-index.html |
